# 

**Published:** 1891-03

**Authors:** 


					﻿Vol. XI.
MARCH, 1891.
NO. 3.
THE
flOMEEOPATHlC pHY^lClA^
A Monthly Journal of Homoeopathic Materia Medica
and Clinical Medicine.
"He is a freeman whom truth makes free, and all are slaves beside."
“Seek the Truth; come whence it may, cost what it will.”
EDITED AND PUBLISHED BY
WALTER ME. JAMES, M. D.,
AND
George H. Clark, M. d.
PHILADELPHIA :
No. 1125 8PRUCK STREET.
LONDON-
ALFRED HEATH & CO., Sole Agents for Great Britain,
114 Ebury Street, Eaton Square.
JtS“Yearly Subscription, $2.50, invariably in advance. Single numbers, 25 cts.
Entered at the Philadelphia Peet-Office aa Second-Claw Mail Matter.
				

